# Exploring and exploiting the genetic variation of Fusarium head blight resistance for genomic-assisted breeding in the elite durum wheat gene pool

**DOI:** 10.1007/s00122-018-3253-9

**Published:** 2018-12-01

**Authors:** Barbara Steiner, Sebastian Michel, Marco Maccaferri, Marc Lemmens, Roberto Tuberosa, Hermann Buerstmayr

**Affiliations:** 1 Department of Agrobiotechnology (IFA-Tulln), Institute of Biotechnology in Plant Production, University of Natural Resources and Life Sciences, Vienna (BOKU), Konrad-Lorenz-Str. 20, 3430 Tulln, Austria; 2 Department of Agricultural and Food Sciences, University of Bologna, 40127 Bologna, Italy

## Abstract

**Key message:**

Genomic selection had a higher selection response for FHB resistance than phenotypic selection, while association mapping identified major QTL on chromosome 3B unaffected by plant height and flowering date.

**Abstract:**

Fusarium head blight (FHB) is one of the most destructive diseases of durum wheat. Hence, minimizing losses in yield, quality and avoiding contamination with mycotoxins are of pivotal importance, as durum wheat is mostly used for human consumption. While growing resistant varieties is the most promising approach for controlling this fungal disease, FHB resistance breeding in durum wheat is hampered by the limited variation in the elite gene pool and difficulties in efficiently combining the numerous small-effect resistance quantitative trait loci (QTL) in the same line. We evaluated an international collection of 228 genotyped durum wheat cultivars for FHB resistance over 3 years to investigate the genetic architecture and potential of genomic-assisted breeding for FHB resistance in durum wheat. Plant height was strongly positively correlated with FHB resistance and led to co-localization of plant height and resistance QTL. Nevertheless, a major QTL on chromosome 3B independent of plant height was identified in the same chromosomal interval as reported for the prominent hexaploid resistance QTL *Fhb1*, though haplotype analysis highlighted the distinctiveness of both QTL. Comparison between phenotypic and genomic selection for FHB resistance revealed a superior prediction ability of the former. However, simulated selection experiments resulted in higher selection responses when using genomic breeding values for early generation selection. An earlier identification of the most promising lines and crossing parents was feasible with a genomic selection index, which suggested a much faster short-term population improvement than previously possible in durum wheat, complementing long-term strategies with exotic resistance donors.

**Electronic supplementary material:**

The online version of this article (10.1007/s00122-018-3253-9) contains supplementary material, which is available to authorized users.

## Introduction

Durum wheat (*Triticum durum* (Desf.)) is susceptible to pathogenic fungi of the *Fusarium* genera such as *Fusarium graminearum and Fusarium culmorum*, which cause Fusarium head blight (FHB), one of the most destructive diseases of wheat worldwide. FHB leads to significant yield losses, but the major concern is the contamination of the crop with mycotoxins. Durum wheat is mostly used for human consumption, and the risk of toxin-contaminated grain entering the food chain is consequently particularly high. During the past few years, FHB on small-grain cereals has significantly increased due to changes in crop management practices, minimum or reduced tillage and intensification of maize in the crop rotation and weather patterns with more humidity and warm temperatures during anthesis (McMullen et al. 2012; Juroszek and von Tiedemann 2015). The increase in demand for pasta products has also led to an expansion of durum wheat production to non-traditional growing regions, like Austria, Germany and France with more humid climatic conditions (UNAFPA 2015). These factors resulted in a higher risk of FHB infections, which is reflected by toxin contamination found in worldwide bread and pasta wheat samples: 57% of 11,022 collected samples were tested positive for the major mycotoxin deoxynivalenol (DON), while 7% exceeded a DON content of 750 μg/kg (Schothorst and van Egmond 2004). In response to this development, the European Commission enacted maximum tolerance levels for DON and other Fusarium toxins in cereals and cereal-based products, including unprocessed durum wheat, bran, wheat flour and pasta (EFSA 2007; Visconti and Pascale 2010).

The development of resistant varieties is the most effective approach for controlling FHB. Resistance breeding in durum wheat has been hampered by the limited genetic variation in its elite gene pool, with most elite durum wheat cultivars being moderately to highly susceptible (Clarke et al. 2010; Miedaner and Longin 2014). For example, in an international collection of 7500 *T. durum* lines comprising accessions from the International Maize and Wheat Improvement Center (CIMMYT) and the International Center for Agricultural Research in the Dry Areas (ICARDA) only five moderately resistant Tunisian lines were detected (Elias et al. 2005; Huhn et al. 2012). A number of studies have thus been conducted for finding sources of resistance in wild or cultivated relatives, e.g. *T. dicoccoides*, *T. dicoccum* and *T. carthlicum* (Buerstmayr et al. 2003; Oliver et al. 2007, 2008; Ruan et al. 2012; Zhang et al. 2014; Zhu et al. 2016), but compared to the reported major FHB resistance QTL in bread wheat (Buerstmayr et al. 2009) only few minor-effect QTL have been identified in tetraploid wheat (Prat et al. 2014; Zhang et al. 2014; Miedaner et al. 2017). The effect of these few QTL is though much smaller in comparison to the strong QTL detected in hexaploid wheat, such as *Fhb1* and *Fhb5* that were discovered in the Chinese cultivar Sumai-3 and derivatives (Anderson et al. 2001; Buerstmayr et al. 2002). Prat et al. (2017) and Zhao et al. (2018) reported recently the successful introgression of the hexaploid resistance QTL *Fhb1*, *Qfhb.ndwp*-*5A* and *Qfhb.ndwp*-*7A* into durum wheat resulting in improved resistance. Nevertheless, durum wheat breeders are often very cautious in introducing ‘exotic’ resistance genes from wild or cultivated relatives into their elite material due to the accompanied linkage drag, which hampers their deployment in modern cultivars.

Despite the lack of highly resistant durum lines, the efficient use of ‘native’ resistance sources present in the elite durum gene pool by combining the numerous small-effect resistance conferring alleles in the same breeding line might be another strategy to achieve acceptable FHB resistance levels. The common practice to pyramid minor resistance QTL by phenotypic selection has been successfully applied in European winter wheat breeding programmes (Kollers et al. 2013) and can nowadays be accelerated by genomic-assisted breeding approaches. Given the decreasing costs of genotyping and sophisticated statistical methods, several new tools are now available to plant breeders for predicting complex traits such as FHB resistance in order to support selection decisions (Crossa et al. 2017). Marker-assisted prediction with few significant marker–trait associations is thereby especially suited for traits controlled by a low number of major QTL, while genomic prediction with a large number of genome-wide distributed markers is capable to additionally target the multitude of minor QTL present in the gene pool (Poland and Rutkoski 2016). Prediction models are thereby trained on a population of phenotyped and genotyped individuals. The estimated marker effects can subsequently be used to predict so-called genomic breeding values for new untested but already genotyped individuals (Whittaker et al. 2000; Meuwissen et al. 2001). The ridge regression best linear unbiased prediction (RR-BLUP) and its equivalent the genomic best linear unbiased prediction (G-BLUP) model are commonly used for practical genomic prediction applications in plant breeding, while the latter model uses a genomic relationship matrix for estimating genomic breeding values directly (Piepho 2009). The application of these predictions for a marker-assisted or genomic selection for FHB resistance breeding has been demonstrated by several studies in hexaploid wheat with the latter showing usually a higher prediction accuracy. Some differences were found depending on the studied FHB resistance trait, resistance sources used and the presence of major QTL as well as the population size that is used for training prediction models (Rutkoski et al. 2012; Arruda et al. 2015, 2016; Jiang et al. 2015; Mirdita et al. 2015; Hoffstetter et al. 2016; Poland and Rutkoski 2016). Low prediction accuracies for FHB resistance (*r* < 0.2) were obtained for marker-assisted selection with few significant markers in an extensive study of 2325 European winter wheat lines where no large effect QTL could be detected. However, employing genomic selection increased prediction accuracies to 0.6 (Mirdita et al. 2015). In addition to increasing the marker number (Jiang et al. 2015; Arruda et al. 2015), exploiting prior information on correlated traits and modelling major QTL as fixed effects have been shown to improve prediction accuracies for FHB related traits in hexaploid wheat (Rutkoski et al. 2012; Arruda et al. 2016), while genetic relationship is also an important driving force of genomic prediction for FHB resistance both in wheat (Hoffstetter et al. 2016) as well as barley (Lorenz et al. 2012; Lorenz and Smith 2015).

Implementation of genomic selection in durum wheat breeding programmes has recently gained interest for yield and quality traits, with prediction abilities obtained from cross-validation varying between *r* = 0.34 and *r* = 0.78 (Crossa et al. 2016; Fiedler et al. 2017; Sukumaran et al. 2018; Haile et al. 2018). In a recent study, Miedaner et al. (2017) detected many small-effect FHB resistance QTL by genome-wide association mapping with a diversity panel of winter durum lines in accordance with previous linkage mapping studies (Prat et al. 2014). Targeting this trait by genomic selection could be a valuable option for improving FHB resistance in durum wheat besides classical recurrent phenotypic selection and give breeders the opportunity to identify lines that combine FHB resistance with other desired traits such as semolina quality in early breeding stages (Fiedler et al. 2017). Such a multi-trait selection has though also to take unfavourable relationships between agronomic traits and FHB severity into account, as it has, for example, been found that shorter plants are often more susceptible to FHB and vice versa (Kollers et al. 2013; Miedaner and Longin 2014; Miedaner et al. 2017; Schulthess et al. 2018). The aims of this study were thus (1) to investigate the genetic architecture of FHB resistance in the elite durum wheat gene pool, (2) assess the accuracy when using phenotypic and genomic selection strategies for the improvement of FHB resistance in the scope of its negative trade-off with plant height and (3) get more insight into possible genomic selection strategies and their response to selection in order to accelerate the genetic improvement of durum wheat.

## Materials and methods

### Plant material and phenotypic data

A diverse population of genetically fixed 228 spring durum lines (*Triticum durum* Desf.) from Northern America, the Mediterranean, Central Europe and Australia as well as from the CIMMYT international durum wheat breeding programme was analysed in this study. The 228 lines were chosen from a larger diversity panel of 269 released varieties and advanced breeding lines, which was assembled by Maccaferri et al. (2006), to obtain a population that was agronomical more homogeneous with regard to plant height and had a narrower range in flowering date (Maccaferri et al. 2011; Liu et al. 2017). The diversity panel was evaluated at the experimental station of the Department of Agrobiotechnology in Tulln (16°04, 16′E, 48°19, 08′N and 177 m above sea level) in 3 years from 2011 to 2013. All three trials were laid out as randomized complete block designs with two replicates, and replicates were sown approximately 1–2 weeks apart resulting in a 1–3 days difference in anthesis between replications. The lines were tested in double rows of 1 m length at 17 cm spacing and inoculated with the DON-producing *Fusarium culmorum* isolate Fc91015. Spray inoculations were performed individually on each plot when 50% of the plants had reached anthesis and were repeated 2 days later. Inoculum was applied with a backpack sprayer at a conidial concentration of 2.5 × 10^4^ ml^−1^, which corresponded to an amount of 100 ml m^−2^ of conidia suspension. A mist irrigation system provided an adequate moisture level for 20 h after each inoculation in order to promote spore germination and fungal infection (Buerstmayr et al. 2002). FHB symptoms were visually scored as percentage of infected spikelets within each plot on days 14, 18, 22 and 26 after the particular plot reached the flowering stage. The area under the disease progress curve (AUDPC) of the four FHB scorings was finally used as an integrated measure of FHB severity. Flowering date records were converted in days after May 1, and plant height was finally measured in centimetre at the end of each season when plants reached the ripening stage.

### Statistical analysis of phenotypic data

Phenotypic analysis for the population of 228 lines was conducted separately for each trial by trait combination using a linear mixed model of the form:

1 $$y_{ik} = \mu + g_{i} + r_{k} + e_{ik}$$

where $y_{ik}$ are the phenotypic records for either FHB severity, plant height or flowering date and $\mu$ is the grand mean. The effect of the *k*th replicate $r_{k}$ was modelled as random, and $e_{ik}$ designates the residual effect with ${\mathbf{e}} \,\sim\,N\left( {0, {\mathbf{I}}\sigma_{e}^{2} } \right)$. The effect $g_{i}$ of the *i*th line was firstly modelled as random to estimate the genetic variance $\sigma_{G}^{2}$ to determine the heritability and subsequently fixed to derive best linear unbiased estimates (BLUEs) in order to avoid a double-shrinkage when training marker-assisted and genomic prediction models. The heritability of each trial by trait combination was determined according to Piepho and Möhring (2007):

2 $$h^{2} = \sigma_{G}^{2} /\left( {\sigma_{G}^{2} + \frac{1}{2}{\text{MVD}}} \right)$$

where $\sigma_{G}^{2}$ is the genetic variance and ${\text{MVD}}$ the mean variance of a difference of the BLUEs. A one-step approach was subsequently employed for the analysis across trials for each trait of interest:

3 $$y_{ijk} = \mu + g_{i} + t_{j} + gt_{ij} + r_{jk} + e_{ijk}$$

where $y_{ijk}$ are again the phenotypic records for each trait, respectively, $\mu$ the grand mean, and $g_{i}$ the effect of the *i*th line with ${\mathbf{g}} \,\sim\, N\left( {0, {\mathbf{I}}\sigma_{g}^{2} } \right)$. The effect of the *j*th trial $t_{j}$ was fixed, while the effect of the *k*th replicate within the *j*th trial $r_{jk}$, the genotype-by-trial interaction $gt_{ij}$ and the residual effect $e_{ijk}$ were modelled as random. The across-year heritability was again estimated by formula (2), and BLUEs for each trait were obtained by modelling a fixed line effect. All phenotypic analyses were conducted using the statistical package ASReml 3 for the R programming environment (R Core Team 2017).

### Genotypic data and population structure

All 228 lines were genotyped using the 90K iSelect wheat SNP assay (Wang et al. 2014). Quality control was applied by filtering out markers with more than 10% missing data and a minor allele frequency < 10%. The random forest algorithm was employed for a chromosome-wise imputation of missing data using the R package *missForest* (Stekhoven and Bühlmann 2012), which led to a set of 12,293 polymorphic markers. The pair-wise correlation between markers was used as an ad hoc measure of linkage disequilibrium, and one marker from each marker pair that had a *r*^2^ = 1.0 was dropped at random to remove strongly correlated predictor variables for genomic predictions giving a final set of 8275 markers. The high-density map for tetraploid wheat by Maccaferri et al. (2015) was employed to determine the chromosomal position of these markers, resulting in an average coverage of one marker every 0.99 cM. SSR markers for the known semi-dwarfing gene *Rht*-*B1* and photoperiodic sensitivity locus *Ppd*-*B1* were additionally included in both marker subsets.

All 228 durum lines were furthermore genotyped for the major resistance QTL *Fhb1* identified in hexaploid wheat (Anderson et al. 2001). Both exons of the pore-forming toxin-like (PFT) gene were analysed which has been found as the causal FHB resistance gene behind *Fhb1* (Rawat et al. 2016). Primer sequences for exon 1 and exon 2 of the PFT gene were as follows:

exon 1: PFT-1F: 5′ATCCAGACCGACCTCAACGT; PFT-1R: 5′CCTTACTCTCCAGCTTGAGAACT; exon 2: PFT-2F: 5′GAAAACAAGCCACGACCCATTC; PFT-2R: 5′TGTCAACCAGCAGGGATACAG.

The *Fhb1* carrier ‘CM-82036’ derived from the well-known resistance source Sumai-3 was used as reference for the sequence comparison at the PFT locus (Buerstmayr et al. 2002). To distinguish between PCR failure and null alleles, a multiplex PCR protocol was employed that simultaneously amplified the respective locus in the *Fhb1* interval and the transcription elongation factor 1 as reference gene in a single PCR with the given primer sequence for the PCR control gene TEF-F: 5′ATGCACCATGAGTCTCTCC; TEF-R: 5′ CTTGATGACACCAACAGCC. In addition, allelic diversity was evaluated for the *Fhb1*-specific marker TaHRC-KASP (Su et al. 2018).

Population structure was analysed by obtaining ancestry estimates based on a non-negative matrix factorization algorithm that has been shown to provide highly accurate estimates in populations with fully inbred lines (Frichot et al. 2014). The optimal number of subpopulations was determined by minimizing a cross-entropy criterion, where marker genotypes of 25% of the lines were initially masked and subsequently predicted in a cross-validation manner with 100 replicates for a range of *K* = 1–10 subpopulations (Frichot et al. 2014). The population structure analysis was thereby based on the R package *LEA* (Frichot and Francois 2015), while a principal components analysis using either the 8275 markers or the BLUEs from the across-trial analysis was also conducted.

### Genome-wide association mapping and marker validation

Firstly, we divided the 228 lines into a mapping population of 180 lines and a validation population of the leftover 48 lines. The entire set of 228 lines was therefore clustered using the partitioning around medoids method with FHB severity, plant height and flowering date from the across-trial analysis as input variables. A predetermined number of 48 clusters were set, and the lines that constituted the medoids were subsequently sampled into the validation population. The clustering aimed to sample a diverse sample of lines representing the entire spectrum of phenotypic values with regard to FHB severity, plant height and flowering date into this validation population. Genome-wide association mapping was afterwards conducted within the mapping population of 180 lines based on a linear mixed model following Yu et al. (2006):

4 $${\mathbf{y}} = {\mathbf{Xb}} + {\mathbf{Ma}} + {\mathbf{Pv}} + {\mathbf{Zg}} + {\mathbf{e}}$$

where ${\mathbf{y}}$ is an *N *× 1 vector of BLUEs obtained in the phenotypic analysis. The fixed effects matrix ${\mathbf{X}}$ and its corresponding vector ${\mathbf{b}}$ modelled the grand mean, while ${\mathbf{a}}$ was a vector for the marker effect and ${\mathbf{M}}$ the incidence matrix of + 1, − 1, and 0 coding for homozygous major, minor and heterozygous, respectively. Population structure and familial relationship were considered by modelling principal components ${\mathbf{v}}$ with a corresponding matrix ${\mathbf{P}}$ as fixed effects as well as integrating an *N* × 1 vector ${\mathbf{g}}$ of random line effects with the genetic variance $\sigma_{G}^{2}$ and ${\mathbf{g}} \,\sim\,N\left( {0, {\mathbf{K}}\sigma_{G}^{2} } \right)$ and random effect design matrix ${\mathbf{Z}}$ into the model. The residual variance $\sigma_{e}^{2}$ finally followed ${\mathbf{e}}\,\sim\,N\left( {0, {\mathbf{I}}\sigma_{e}^{2} } \right)$. The necessary genomic relationship matrix ${\mathbf{K}}$ was computed according to Endelman and Jannink (2012):

5 $${\mathbf{K}} = {\mathbf{WW}}^{\text{T}} /2\varSigma \left( {p_{k} - 1} \right)p_{k}$$

where ${\mathbf{W}}$ is a centred *N* × *M* marker matrix of the *i* lines with $W_{ik} = Z_{ik} + 1 - 2p_{k}$ and $p_{k}$ being the allele frequency at the *k*th locus. A preliminary analysis revealed though no appreciable effect of modelling principal components (Fig. S1) with the given population structure (Fig. S4); thus, for all subsequent genome-wide association mapping analyses, model (4) was reduced to:

6 $${\mathbf{y}} = {\mathbf{Xb}} + {\mathbf{Ma}} + {\mathbf{Zg}} + {\mathbf{e}}$$

retaining the above-described designations and assumptions. A first analysis was carried out with all 180 lines in the mapping population with the BLUEs from the across-trial analysis (3), and marker–trait associations that exceeded an exploratory threshold of − log_10_*p* value = 3 were declared significant, while the most promising marker per locus was chosen by a stepwise regression based on Akaike’s information criterion. A permutation test was subsequently carried out by randomly reshuffling the phenotypes 1000 times in order to obtain a null distribution that was used to determine an experiment-wise significance threshold with *α* ≥ 5% for each trait. The Bonferroni correction resulted in a threshold of − log_10_*p* value = 5.22 at *α* ≥ 5% and is reported as the most conservative significance threshold in this study. Finally, all markers passing the exploratory significance threshold were fitted simultaneously in a linear model for each trait separately in the decreasing order of their − log_10_*p* value beginning with the strongest marker–trait association (Würschum et al. 2015). The sum of squares of an analysis of variance (ANOVA) from the linear model was then used to compute the proportion of explained genetic variance by $\rho_{G} \left( \% \right) = 100 \times \left( {{\text{SS}}_{\text{M}} /{\text{SS}}_{\text{Total}} } \right) \times \left( {h^{2} } \right)^{ - 1}$ with ${\text{SS}}_{\text{M}}$ being the sum of squares for the individual marker, ${\text{SS}}_{\text{Total}}$ the total sum of squares and $(h^{2} )^{ - 1}$ the inverse of the heritability. Genome-wide association mapping was conducted with the R package *sommer* (Covarrubias-Pazaran 2016).

### Comparison of marker-assisted, genomic and phenotypic prediction

Following genome-wide association mapping, there was some interest in comparing the potential of marker-assisted selection based on the found marker–trait associations with genomic and phenotypic selection. Therefore, a training population of *N* = 160 lines was sampled from the mapping population to predict the performance of *V* = 40 lines sampled from the validation population in a cross-validation manner. The sampling was repeated 50 times, and prediction models were trained with data from one trial to predict the validation population in the two other trials at a time. Hence, a cross-validation that sampled both genotypes and environments was applied for the comparison of the different prediction models. Marker-assisted selection was conducted by using the three most significant markers for each trait with marker effects being estimated by a ridge regression best linear unbiased prediction (RR-BLUP) model for each trait separately:

7 $${\mathbf{y}} = {\mathbf{Xb}} + {\mathbf{Zu}} + {\mathbf{e}}$$

where ${\mathbf{y}}$ is a *N* × 1 vector of BLUEs from the phenotypic analysis, ${\mathbf{b}}$ is a vector of fixed effects and ${\mathbf{X}}$ its corresponding design matrix. ${\mathbf{Z}}$ is a *N* × *M* matrix that contained the marker coding for the M candidate markers of each trait, respectively, and the random marker effects were assumed to follow a normal distribution ${\mathbf{u}} \,\sim\, N\left( {0, {\mathbf{I}}\sigma_{u}^{2} } \right)$ with variance $\sigma_{u}^{2}$ and ${\mathbf{e}} \,\sim\,N\left( {0, {\mathbf{I}}\sigma_{e}^{2} } \right)$. Genomic breeding values for a genomic-based selection were obtained from a genomic best linear unbiased prediction (G-BLUP) model:

8 $${\mathbf{y}} = {\mathbf{Xb}} + {\mathbf{Zg}} + {\mathbf{e}}$$

where ${\mathbf{y}}$ is again the vector of phenotypic records, while ${\mathbf{g}}$ is an (*N* + *V*) × 1 vector of line effects with the genetic variance $\sigma_{G}^{2}$ and ${\mathbf{g}} \sim N\left( {0, {\mathbf{K}}\sigma_{G}^{2} } \right)$ as well as the random effect design matrix ${\mathbf{Z}}$. The fixed effect matrix ${\mathbf{X}}$ and the corresponding vector ${\mathbf{b}}$ modelled merely the grand mean in the G-BLUP model, whereas in the weighted genomic best linear unbiased prediction (W-BLUP) model for an enhanced genomic-based selection also contained a fixed effect for the most promising candidate marker from genome-wide association mapping in the mapping population (Bernardo 2014; Zhao et al. 2014). In these cases, the genomic breeding value of each line was estimated by:

9 $${\text{GEBV}}_{i} = g_{i} + \mathop \sum \limits_{j = 1}^{n} x_{ij} u_{j}$$

with $g_{i}$ being the random genetic effect of the *i*th line, $u_{j}$ the estimated effect of the *j*th marker, and $x_{ij}$ being the marker allele of the *i*th line at the *j*th marker. Model (8) was also employed to estimate kinship enhanced phenotypic breeding values (K-BLUP) assuming that phenotypic records of the selection candidates in the validation population were already available for a genomic-assisted selection (Michel et al. 2017). The prediction ability of all genomic models and phenotypic selection was assessed by the correlation between breeding values and phenotypic records with BLUEs obtained from the single trial analysis by model (1). Ridge regression models were fitted with *rrBLUP* (Endelman 2011), while all other models in this section were based on *sommer* (Covarrubias-Pazaran 2016) for R (R Core Team 2017).

### Multi-trait prediction models and genomic selection indices

A further option to improve the accuracy of genomic breeding values would be the application of phenotypic imputation (Jia and Jannink 2012) or trait-assisted selection (Fernandes et al. 2017) with multi-trait genomic prediction models containing pre-existing information of one or several correlated traits that could be measured earlier, easier or more cost-efficient than the main trait of interest. Hence, the genomic relationship matrix ${\mathbf{K}}$ was utilized again for fitting a multi-trait mixed linear model, which contained plant height or flowering date as well as both as correlated traits and the FHB severity as main trait of interest:

10 $${\mathbf{y}}_{\varvec{t}} = {\mathbf{X}}_{\varvec{t}} {\mathbf{b}}_{\varvec{t}} + {\mathbf{M}}_{\varvec{t}} {\mathbf{g}}_{\varvec{t}} + {\mathbf{e}}_{\varvec{t}}$$

where ${\mathbf{y}}_{\varvec{t}}$ is a *N* × *t* vector of BLUEs for t traits obtained in the phenotypic analysis, ${\mathbf{g}}_{\varvec{t}}$ is the vector of *N* × *t* line effects with the corresponding random effect design matrix ${\mathbf{M}}_{\varvec{t}}$ and ${\mathbf{g}}_{\varvec{t}} \,\sim\, {\text{MVN(}}0, \sum_{g} \otimes {\mathbf{K}})$ with the completely unstructured variance–covariance matrix $\sum_{g}$ of the form:

11 $$\left( {\begin{array}{*{20}l} {\sigma_{{g_{\text{FHB}} }}^{2} } \hfill & {\sigma_{{g_{\text{FHB;PH}} }} } \hfill & {\sigma_{{g_{\text{FHB;FD}} }} } \hfill \\ {\sigma_{{g_{\text{FHB;PH}} }} } \hfill & {\sigma_{{g_{\text{PH}} }}^{2} } \hfill & {\sigma_{{g_{\text{PH;FD}} }} } \hfill \\ {\sigma_{{g_{\text{FHB;FD}} }} } \hfill & {\sigma_{{g_{\text{PH;FD}} }} } \hfill & {\sigma_{{g_{\text{FD}} }}^{2} } \hfill \\ \end{array} } \right)$$

where $\sigma_{{g_{\text{FHB}} }}^{2}$, $\sigma_{{g_{\text{PH}} }}^{2}$ and $\sigma_{{g_{\text{FD}} }}^{2}$ are the genetic variance of the FHB severity, plant height and flowering date, respectively, and values on the off-diagonal genetic represent covariances between the traits. The variance of the residual effect followed ${\mathbf{e}}_{\varvec{t}} \sim {\text{MVN}}\left( {0, \sum_{e} \otimes {\mathbf{I}}_{\varvec{N}} } \right)$ where ${\mathbf{I}}_{\varvec{N}}$ is an identity matrix of dimension *N* × N and $\sum_{e}$ the completely unstructured variance–covariance matrix for the residual effect analogues to (11) though with residual variances and covariance between traits. The fixed effect part ${\mathbf{X}}_{\varvec{t}} {\mathbf{b}}_{\varvec{t}}$ of model included a fixed effect ${\mathbf{b}}_{\varvec{t}}$ with three levels for the respective traits. The matrices $\sum_{g}$ and $\sum_{e}$ were accordingly modified if plant height, flowering date or both traits were assumed to be known beforehand for lines in the validation population. A training population of *N* = 160 lines was again sampled from the mapping population to predict the performance of *V* = 40 lines sampled from the validation population for an across-trial prediction as beforehand, while the pre-existing information about plant height and flowering date came only from the trial that was employed for training the multi-trait prediction models.

The obtained genomic breeding values were employed to conduct a simultaneous selection for multiple agronomic traits and FHB resistance, which might often be challenging especially due to the frequently observed negative correlation between FHB severity and plant height. A genomic selection index was calculated by utilizing the weights of a desired gain index that was modified to a restriction index with three traits to account for this negative trade-off (Pesek and Baker 1969, 1970):

12 $${\mathbf{b}} = {\mathbf{G}}^{ - 1} {\mathbf{a}}$$

where ${\mathbf{b}}$ are the index weights, ${\mathbf{a}}$ is a vector of desired gains given by ${\mathbf{a}} = \left( {a_{\text{FHB}} ,a_{\text{PH}} ,a_{\text{FD}} } \right)^{\text{T}}$, and ${\mathbf{G}}^{ - 1}$ is the inverse of the genotypic variance–covariance matrix between the three traits estimated by the multi-trait model (12). Aiming to improve FHB resistance and holding the other two traits constant, i.e. identifying lines that have a high FHB resistance relative to their plant height and heading date, the desired gains for FHB severity, plant height and flowering date were $a_{\text{FHB}} = 1$, $a_{\text{PH}} = 0$ and $a_{\text{FD}} = 0$, respectively. The matrix ${\mathbf{G}}$ was alternatively derived from the Pearson correlation between the genomic breeding values of the three involved traits in order to investigate an alternative to the calculation of the variance–covariance matrix from a multi-trait model, which has a high computational demand. Both genomic selection indices with a matrix ${\mathbf{G}}$ derived either from a multi-trait model or the Pearson correlation between genomic breeding values were furthermore calculated based on all lines in the respective training and validation population, which was compared with a variant that calculates genomic selection indices solely with lines in the validation population that are relevant for pending selection decisions. The ability to predict FHB severity was assessed by the correlation between multi-trait predictions and genomic selection indices with the observed phenotypic values obtained from the across-trial analysis, while the relationship of these predictors with plant height and flowering date was also recorded to measure the correlated response to selection. Linear mixed models for multi-trait prediction and genomic selection indices were again fitted with the R package *sommer* (Covarrubias-Pazaran 2016).

### Phenotypic and genomic selection strategies

**Fig. 1:**
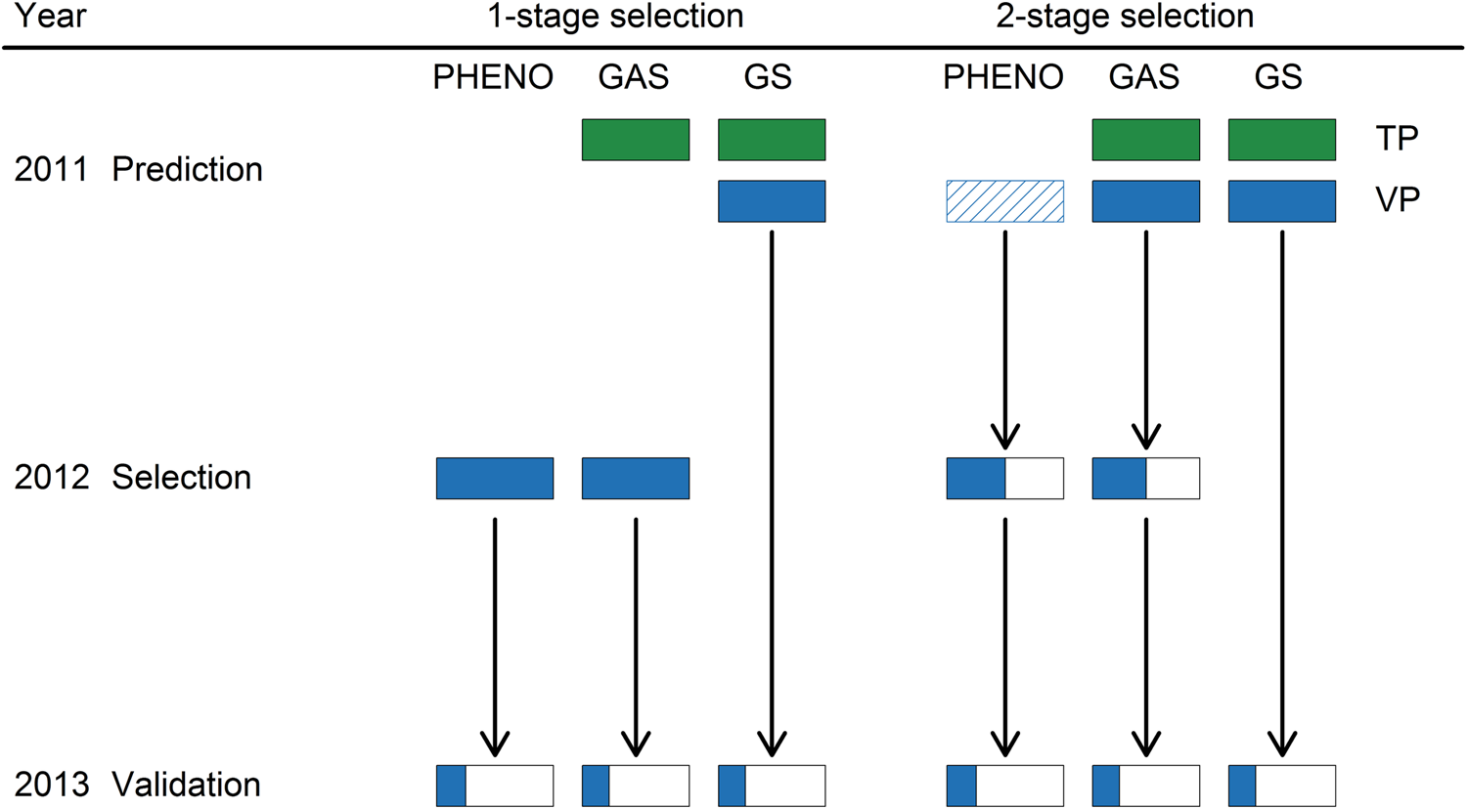
One-stage and two-stage selection strategies using phenotypic (PHENO), genomic-assisted (GAS) or genomic-based (GS) selection using the available information from the validation population (VP) as well as from the training population (TP). Phenotypic selection in the first year of a two-stage selection strategy was based only on flowering date or plant height, while in the other methods selection was conducted with genomic breeding values for all involved traits

Phenotypic and genomics-assisted selection for FHB resistance was subsequently compared in several simulated selection experiments featuring three selection strategies that might be applied in a practical breeding programme (Fig. 1):

1. One-stage single-trait selection with extensive disease phenotyping, where all selection candidates are either phenotyped in disease nurseries or genomically selected without phenotyping.
2. Two-stage single-trait selection, where half of the selection candidates are discarded in the first stage, while testing in disease nurseries and final selection decisions are conducted among the preselected candidates.
3. Two-stage multi-trait selection for all three investigated traits (FHB, plant height and flowering date) simultaneously, where half of the selection candidates are discarded in the first stage either by phenotypic culling or genomic index selection, and preselected candidates are retested in disease nurseries for conducting final selection decisions.

A small-scale durum breeding programme was assumed for the purpose of this study, where the budget allows assessing FHB resistance only in one artificially inoculated field trial each year. Separate training population and selection populations were 50 times sampled for all simulated selection experiments, where the former comprised, as in the assessment of the prediction abilities, *N* = 160 lines from the mapping population and the latter *V* = 40 lines that were sampled from the validation population. It should be mentioned that these numbers were seen as appropriate given the exploratory nature of this study, although training and validation population sizes are generally much larger in applied breeding programmes.

Genomic-based selection relied on a G-BLUP model with a training population that was phenotyped in one trial/year, which was designated as the prediction year. Phenotypic selection was on the other hand based on phenotypic data of the selection candidates from a second trial/year designated as the selection year. Genomic-assisted selection was finally based on the combination of data obtained in the prediction and selection year, where selection was conducted with kinship enhanced phenotypic breeding values derived from model (10) with a fixed trial/year effect. FHB resistance information about lines in the selection population was assumed absent in the prediction year, while it seemed reasonable to assume that phenotypic information about plant height and flowering date would already be available in early generations when selection candidates are genotyped.

Given this framework, several basic approaches were initially compared for the one-stage single-trait selection with extensive disease phenotyping:

(1.1) Phenotypic selection and genomic-assisted selection among all 40 selection candidates.
(1.2) Genomic-based and enhanced genomic-based selection among all 40 selection candidates, where the most promising marker for FHB severity was included as fixed effect into the latter.

Moreover, the two-stage single-trait selection strategy aimed to reflect a typical scenario in many line breeding programmes where genomic selection is conducted in parallel to preliminary or observation yield trials (Michel et al. 2017; Gaynor et al. 2017). Phenotypic information about FHB severity is often not available in this early stage of selection, and phenotyping for FHB resistance is prolonged to advanced breeding stages where the breeding material is screened in disease nurseries in parallel to multi-environment yield trials. Selection has, however, to be conducted in both stages of variety development in such cases due to the limited resources of breeding programmes, and to simulate this breeding scheme, lines were again sampled in a training (160) and selection population (40) with individual trials 2011–2013 being designated as prediction, selection and validation year. Three approaches that involve either none or a preselection by culling were compared for this strategy:

(2.1) Phenotypic selection with culling in the first stage, i.e. the prediction year, where no FHB resistance information for lines in the selection population was available yet. Among the 40 selection candidates, the 10 earliest and latest lines, respectively, were culled and the leftover 20 lines were ‘advanced’ for FHB resistance phenotyping in the selection year.
(2.2) Genomic-assisted selection with culling of the 20 most susceptible selection candidates according to genomic-based breeding values, followed by genomic-assisted selection among the preselected lines in the selection year.
(2.3) Genomic-based and enhanced genomic-based selection among all selection candidates as described in 1.2).

The flowering date served as a representative ‘neutral’ trait as it did not display a strong correlation with FHB severity in the investigated durum wheat population. Earliness is furthermore a general aim in many durum breeding programmes aside from many other traits like yellowness, protein content or yield components that might play an important role in early generation selection.

The simultaneous selection for multiple agronomic traits and FHB resistance could accordingly be challenging, especially due to the often-observed negative correlation between FHB severity and plant height. Hence, the merit of genomic selection indices that took this issue into consideration was investigated in the framework of the two-stage multi-trait selection strategy by:

(3.1) Phenotypic selection with culling in the first stage, i.e. the prediction year, where no FHB resistance information for lines in the selection population was available yet. Among the 40 selection candidates, the 10 smallest and tallest lines, respectively, were again culled and the leftover 20 lines were ‘advanced’ for FHB resistance phenotyping in the selection year.
(3.2) Genomic-assisted index selection with culling of the 20 most susceptible selection candidates according to a genomic-based selection index, followed by genomic-assisted index selection among the preselected lines in the selection year.
(3.3) Genomic-based and enhanced genomic-based selection with a genomic selection index among all selection candidates as described above.

The culling of the smallest and tallest lines was, as a breeder would expect that lines with a very short stature will show also a strong susceptibility to FHB, while tall-statured plants will most likely be more resistance to FHB but on the other hand possess less tolerance against lodging. Genomic index selection was conducted with the previous described restriction index (12) aiming to improve FHB resistance and holding the other two traits constant, while phenotypic records for plant height and flowering date were again assumed to be already available in the prediction year used for model training. The final selection decision for all selection strategies was undertaken by selecting the 6–20 most resistant lines identified by each of the above-described approaches, in which the left-out third year, i.e. the validation year, was employed to determine the response to selection by:

13 $$R_{{{\text{Rel}}\,i}} = \left( {\mu_{{{\text{Sel}}\,i}} - \mu_{i} } \right)/\mu_{i}$$

where $R_{{{\text{Rel}}\,i}}$ is the relative response to selection for the *i*th trait, $\mu_{i}$ is the population mean of the selection population in the validation year, and $\mu_{{{\text{Sel}}\,i}}$ is the mean of the selected population of 6–20 best performing lines. Although selection was based only on FHB severity, the indirect response to selection for plant height and flowering date was also assessed. Selection was furthermore conducted for all combinations of trials/years, thus each trial/year served once as training, selection and validation year within each of the 50 replicates of random sampling.

## Results

### Quantitative genetic parameters and trait correlations

A medium to high heritability was obtained for all investigated traits both in the analysis of individual trials and in the across-trial analysis highlighting a sufficient data quality for further phenotypic and genomic analyses (Table 1). Following the respective heritability estimates, the correlation among trials was highest for plant height (*r* = 0.79–0.82), followed by flowering date (*r* = 0.51–0.59), and lowest for FHB severity (*r* = 0.44–0.54) (Table S1). A comparatively narrow range was observed for flowering date given a diversity panel that was constructed from a worldwide collection of durum lines, which was, however, expected due to the preselection of lines with regard to agronomically homogeneous properties under Central European conditions. The range for plant height in the retained lines was larger and had a maximum of 90 cm due to the set culling level when preselecting lines for further analyses in this study. A large genetic variance was estimated using the AUDPC as FHB severity measure, which was four times larger than the genotype-by-trial interaction variance though smaller than the residual variance (Table 1) suggesting the need of FHB experiments to be conducted in replicates as well as several environments to obtain high quality phenotypic data (Fuentes et al. 2005).

**Table 1:** Mean, range, variance components and heritability of FHB severity (AUDPC), plant height (cm) and flowering date (days after May 1) for the individual trials and across the entire trial series 2011–2013

Trait	Trial	$\sigma_{G}^{2}$	$\sigma_{GT}^{2}$	$\sigma_{e}^{2}$	*h* ^*2*^	Min	Mean	Max
FHB severity	2011	27,192		36,211	0.60	225	811	1303
	2012	9306		13,954	0.57	358	725	1030
	2013	23,745		35,087	0.57	207	783	1180
	Series	16,501	4859	26,125	0.79	309	739	1094
Plant height	2011	26.00		9.86	0.84	55	66	90
	2012	25.40		17.28	0.75	55	68	90
	2013	21.85		8.47	0.84	53	68	90
	Series	25.29	0.00	10.81	0.93	55	67	89
Flowering date	2011	0.82		1.56	0.51	26	30	33
	2012	1.61		2.68	0.54	30	33	38
	2013	2.64		1.59	0.77	38	41	46
	Series	1.27	0.35	1.79	0.81	32	34	38

Genotypic variance ($\sigma_{G}^{2}$), genotype × trial interaction variance ($\sigma_{GT}^{2}$), residual variance ($\sigma_{e}^{2}$), heritability (*h*^*2*^)

The correlation between the AUDPC and the last FHB scoring was high (*r* = 0.93) (Fig. S2A). The heritability of the latter trait was though with *h*^2^ = 0.73 slightly lower than the heritability of the AUDPC (*h*^2^ = 0.79), which further underlined the merit of repeated scorings during the field season. Despite the fact that the range of FHB severity was rather large, no resistant lines could be identified as their performance varied between medium to highly susceptible. Lines with Canadian origin displayed thereby the best performance, but also showed the highest plant height under Central European conditions (Fig. S3). They were directly followed by lines developed in the USA whose plant height was on average lower, whereas a large range was observed for the other origins though with strong similarity in the average plant height across origins. Considering the entire panel, a negligible correlation was found between flowering date and FHB severity based on BLUEs from the across-trial analysis (*r* = 0.09 with *p* value = 0.175) (Fig. S2B), while the correlation between plant height and FHB severity was more pronounced for the entire panel (*r* = − 0.29 with *p* value < 0.001) though dependant on the presence of the semi-dwarfing allele at the *Rht*-*B1* locus (Fig. S2C).

### Phenotypic and genotypic population structure

**Fig. 2:**
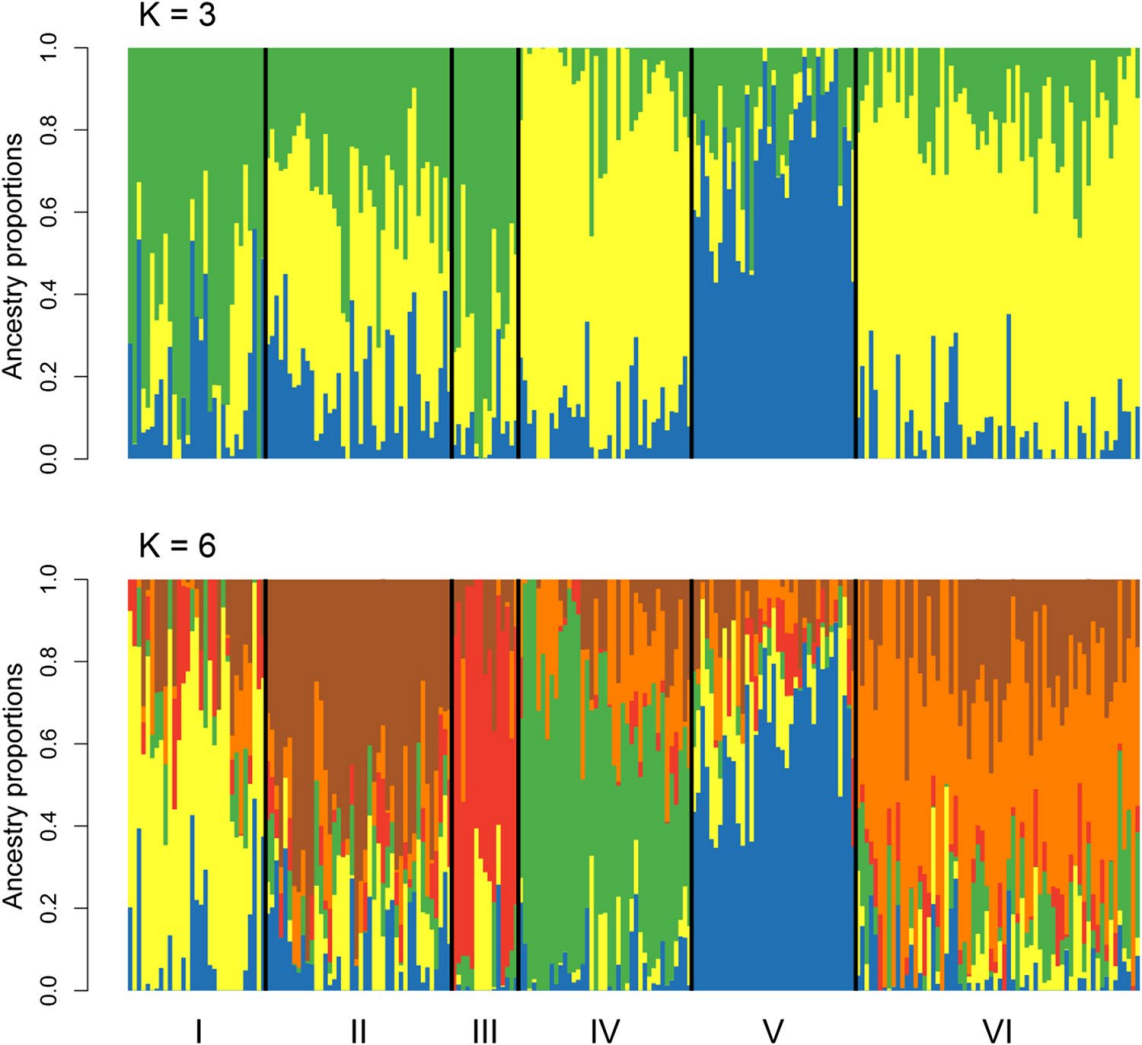
Population structure inferred by estimating individual ancestry coefficients with *K* = 3 (cross-entropy = 0.527) and *K* = 6 (cross-entropy = 0.486) subpopulations. Lines in subpopulations I–IV and VI contained mostly lines with Mediterranean origin, while subpopulation V was dominated by lines with North American, Western and Central European origin

Principal component analysis did not reveal a clear population structure neither on the phenotypic level based on Euclidean distance between standardized phenotypic data for plant height, flowering date and FHB severity nor genotypically based on genome-wide distributed markers (Fig. S4). Lines from ICARDA and CIMMYT were closely related to each other, highlighting most likely the close collaboration and germplasm exchange between these institutions. Lines with other origins were more widely spread, and especially lines from Italy could be found throughout the target genetic space. Estimation of ancestry coefficients showed accordingly an admixture between origins, while in the STUCTURE-like analysis (Frichot et al. 2014) with three subpopulations, two groups were again dominated by lines from CIMMYT and ICARDA, and the other group contained most of the lines with origin in Canada, the USA, Austria and France (Fig. 2). This differentiation was retained when refining the resolution to six subpopulations, where lines in subpopulations I–IV and VI contained mostly lines with Mediterranean origin and subpopulation V lines with North American, Western and Central European countries of origin. The latter subpopulation showed thereby on average the lowest FHB severity (AUDPC = 642), subpopulation IV the highest (AUDPC = 808), and the other subpopulations were on average rather equal (AUDPC = 730–749). A Mantel test of the phenotypic and genomic relationship matrix revealed a significant correlation of both matrices with *r* = 0.31, hence lines that were sampled into the validation population by the partitioning around medoids clustering represented the whole range of phenotypes in the panel were equally spread over the entire target genetic space and came from all designated origins (Fig. S4). Therefore, they represented the worldwide durum germplasm collection well and were regarded as suitable for validating marker–trait associations found by genome-wide association mapping.

### Genome-wide association mapping and marker validation

**Fig. 3:**
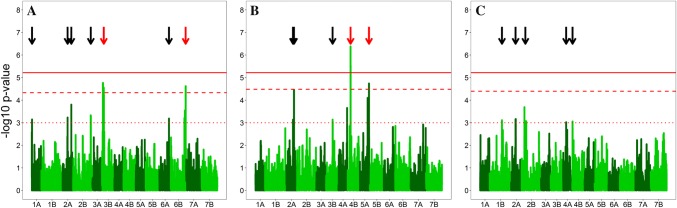
Manhatten plots for genome-wide association mapping of FHB severity (**a**), plant height (**b**) and flowering date (**c**) where the high-confidence marker–trait associations are highlighted with red arrows. The exploratory significance threshold of − log_10_*p* value = 3 is indicated by the dotted line, the significance threshold determined by permutation by the dashed line, and the Bonferroni corrected significance level by the solid line

Seventeen marker–trait associations for FHB severity, plant height and date of anthesis were detected above the exploratory threshold of − log_10_*p* value = 3 using the BLUEs from across-trial analysis of 180 durum wheat lines and 8275 markers (Table 2, Table S2). A clear marker–trait association was found for the semi-dwarfing QTL *Rht*-*B1* on chromosome 4B (Fig. 3) as well as one additional locus for plant height on chromosome 5A. Both of these detected QTL passed the significance threshold determined by the permutation test and could furthermore be validated using the 48 lines that were entirely left out from the mapping process (Table S3). The marker–trait association with *Rht*-*B1* was additionally the only one that passed the conservative Bonferroni corrected significance threshold in the entire dataset. Five markers on chromosomes 1B, 2A, 2B, 4A and 4B could be identified for flowering date, which had minimal effects in the mapping as well as in the validation population and did, moreover, only pass the exploratory threshold. It was evident that all plant height associated markers had an impact on FHB resistance. Accordingly, the semi-dwarfing alleles at *Rht*-*B1* and the 5A locus reduced plant height, but had at the same time a strong negative influence on FHB resistance. Their allele frequencies were anyhow high in the diversity panel of released varieties and breeding lines (Table 2), which could also be expected in modern elite germplasm due to necessary increase in resistance to lodging in durum wheat breeding (De Vita et al. 2007).

**Table 2:** Chromosomal position, QTL detection frequency and additive effect of markers associated with Fusarium head blight severity (FHB), plant height (PH) and flowering date (FD) in the mapping and validation population

Marker	Chrom.	Position	Trait^a^	*p* ^‡^	Add. eff. population^b^			FA^c^	*ρ*_G_^d^
					FHB	PH	FD		
IWB72690	1A	1.7	FHB	3.15	− 23.8	− 1.3	− 0.2	0.29	9.7
IWB36357	1B	82.7	FD	3.11	18.3	0.6	− 0.5	0.89	5.3
IWB32396	2A	101.6	FD	3.16	− 47.9	0.3	− 0.8	0.86	19.1
IWB46663	2A	109	FHB	3.23	− 67.6	0.9	0.2	0.32	1.8
IWB44254	2A	158.7	PH	3.14	29.2	− 1.1	0.4	0.88	0.5
IWB36028	2A	181.2	PH	4.47	70.2	− 2.6	0.0	0.87	4.6
IWB24986	2A	197.6	FHB	3.8	− 84.9	1.4	0.1	0.13	5.7
IWB40861	2B	53.4	FD	3.07	21.5	− 0.9	− 0.6	0.57	11.9
IWB5439	2B	172.3	FHB	3.3	− 52.7	1.6	0.1	0.26	4.9
IWB64968	3B	8.0	FHB	4.55	− 65.8	0.8	0.0	0.35	17.8
IWB36517	3B	92.2	PH	3.14	8.9	− 1.4	− 0.1	0.24	7.8
IWB24360	4A	105.5	FD	3.02	− 7.3	− 0.8	− 0.7	0.11	5.4
IWB74227	4B	2.8	FD	3.06	42.9	− 1.7	− 0.7	0.87	10.3
IWB56078	4B	32.9	PH	5.56	32.1	− 2.1	0.0	0.81	11.3
IWA1670	5A	188.9	PH	4.74	49.7	− 2.3	0.3	0.81	8.4
IWB70133	6A	124.8	FHB	3.19	− 42.3	− 0.4	− 0.4	0.83	3.8
IWB66697	6B	155.1	FHB	4.62	− 64.9	1.5	0.1	0.11	9.0

^‡^− log_10_(*p* value) with high-confidence marker–trait associations being underlined

^a^The trait for which the marker was detected in the mapping population

^b^Additive effect in the entire population of 228 lines

^c^Frequency of lines carrying the favourable allele (%)

^d^Explained genetic variance (%)

Seven loci were found to improve FHB resistance in the international durum wheat collection at hand that were mapped to positions on chromosomes 1A, 2A, 2B, 3B, 6A and 6B. Merely, two marker–trait associations on chromosomes 3B and 6B were retained when filtering according to the permutation test based significance threshold of − log_10_*p* value = 4.33 for high-confidence markers. Both had pronounced additive effects in the mapping population, which could also be confirmed for the 3B QTL in the validation population (Table S3). The 3B QTL-linked marker IWB64968 had a minimal effect on plant height (additive effect of 0.8) and no effect on flowering date, in the entire population, whereas the 6B QTL-associated marker IWB66697 had a pronounced positive influence on plant height (additive effect of 1.5) analysing all 228 lines. The contradicting results for the 6B FHB resistance QTL on plant height between the mapping and validating population were most likely due to the low resistance allele frequency of 11% and might be the result of a sampling effect (Table S3).

The major resistance QTL in this durum germplasm mapped to chromosome 3B between the SSR markers X*barc133* and X*gwm493* that designates the confidence interval of the prominent resistance QTL *Fhb1* in hexaploid wheat (Buerstmayr et al. 2009; Maccaferri et al. 2015). *Fhb1* has been partly elucidated, and a pore-forming toxin-like gene (PFT) was identified to confer resistance against fungal spread (Rawat et al. 2016). Consequently, to investigate the presence of *Fhb1* in durum wheat the entire panel was analysed for the PFT gene and the *Fhb1*-specific marker TaHRC-KASP (Su et al. 2018) (Table S2). All durum lines carried the non-*Fhb1* allele for the marker TaHRC-KASP, and all but two lines exhibited the null allele for the PFT gene. Surprisingly only the susceptible Syrian landrace variety ‘Haurani’ carried the same functional PFT allele as the *Fhb1* reference ‘CM-82036’. The sequence comparison between Haurani and ‘CM-82036’ revealed a single SNP resulting in a synonymous mutation (C < T; S168S), whereas for the second PFT carrier, the susceptible French durum variety Exeldur, a missense mutation in the second exon of the gene was detected (A < T; M140K). In conclusion, the detected FHB resistance QTL on chromosome 3B in durum wheat is dissimilar to *Fhb1,* and only one susceptible line possessed the functional PFT gene. Nevertheless, the found marker–trait association on chromosome 3B deserves further attention due to the generally narrow genetic variation of FHB resistance in durum and might be among others a useful resource to support genomic breeding strategies.

### Single-trait and multi-trait genomic prediction

**Fig. 4:**
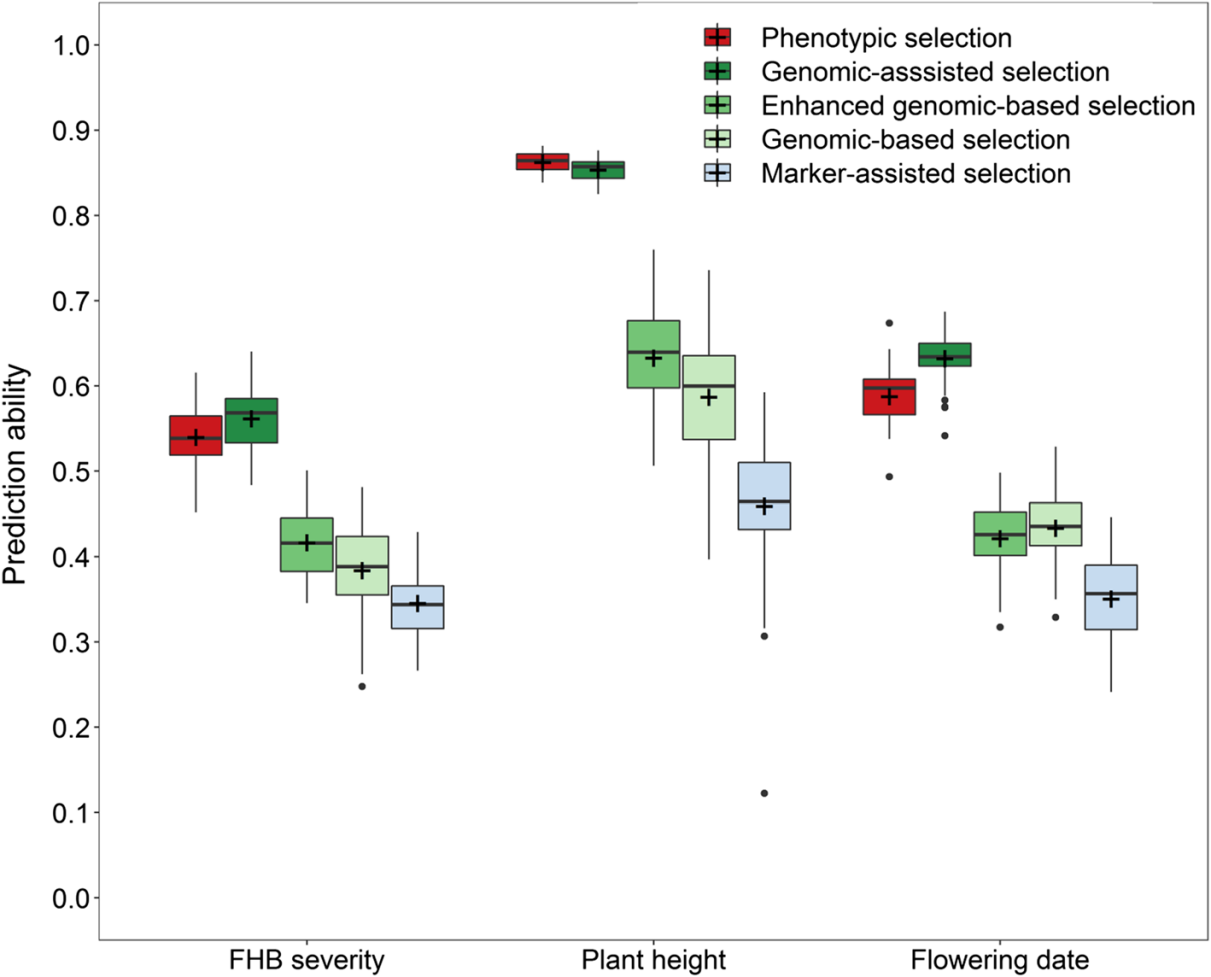
Comparison between phenotypic, genomic-assisted, marker-assisted, genomic-based and enhanced genomic-based selection with upweighting major QTL using the mapping population for model training and predicting the validation population in a cross-validation across trials

The detected marker–trait associations for FHB severity and the other traits were accordingly tested for their merit in a marker-assisted selection, which was parallelly compared with phenotypic and genomic selection using the entire set of 8275 genome-wide distributed markers. Predicting the validation population that was left out from the marker discovery was feasible using the previously found marker–trait associations, although genomic selection was clearly superior to marker-assisted selection for most traits especially when enhanced by modelling one major QTL as fixed effect (Fig. 4). For this purpose, a larger weight was given to the identified FHB resistance marker on chromosome 3B, while the 5A marker was chosen for plant height due to its equal or even larger effect than the *Rht*-*B1* locus among the high-confidence markers, and the 2A marker for flowering date as it was the most reliable marker–trait association for this trait according to the p value. The relative advantage of enhanced genomic-based versus marker-assisted selection in terms of prediction ability was accordingly 21% for FHB severity, 38% for plant height and 20% for flowering date. Both approaches were, however, far inferior to phenotypic selection based on BLUEs, while using the marker information to model relationship between lines in a genomic-assisted selection gave some increase in prediction ability of 4% for FHB severity and 8% for flowering date but had no effect on the prediction of plant height.

**Fig. 5:**
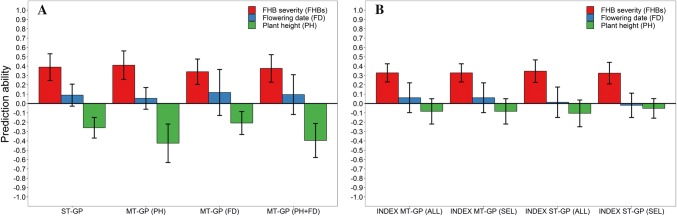
Comparison between single-trait (ST-GP) and multi-trait predictions (MT-GP) using prior information about plant height (PH) and flowering trait (FD) when estimating genomic breeding values for FHB severity (**a**) with data from the across-trial analysis. Genomic index selection for FHB severity based either on a variance–covariance matrix involving all lines (ALL) or merely the selection candidates (SEL) (**b**)

Aside from upweighting major QTL in the prediction of FHB severity, phenotypic imputation or trait-assisted selection with prior information about plant height and flowering trait was tested as a convenient option to increase the prediction ability as both traits are generally early available in a breeding programme. The employed multivariate model could accordingly increase the prediction ability for FHB severity from *r* = 0.39 to *r* = 0.41 (Fig. 5a). The negative trade-off between genomic breeding values for FHB severity and observed phenotypic values, i.e. BLUEs for plant height, was though strongly inflated from *r* = − 0.26 to *r* = − 0.43 by this method, which could be partially compensated by employing a genomic selection index with restrictions (Fig. 5b). A negligible difference could be observed between when the necessary variance–covariance matrix for calculating the index was derived by involving all lines or merely the selection candidates. However, the negative trade-off could only partially be compensated with the parameters obtained from a multi-trait model (*r* = − 0.08). Using a matrix based on Pearson correlations between genomic breeding values enabled on the other hand a stronger adjustment for plant height (*r* = − 0.05) that was though, as expected, accompanied by a reduction in prediction ability for FHB severity (*r* = 0.33).

### Genomic breeding strategies

**Fig. 6:**
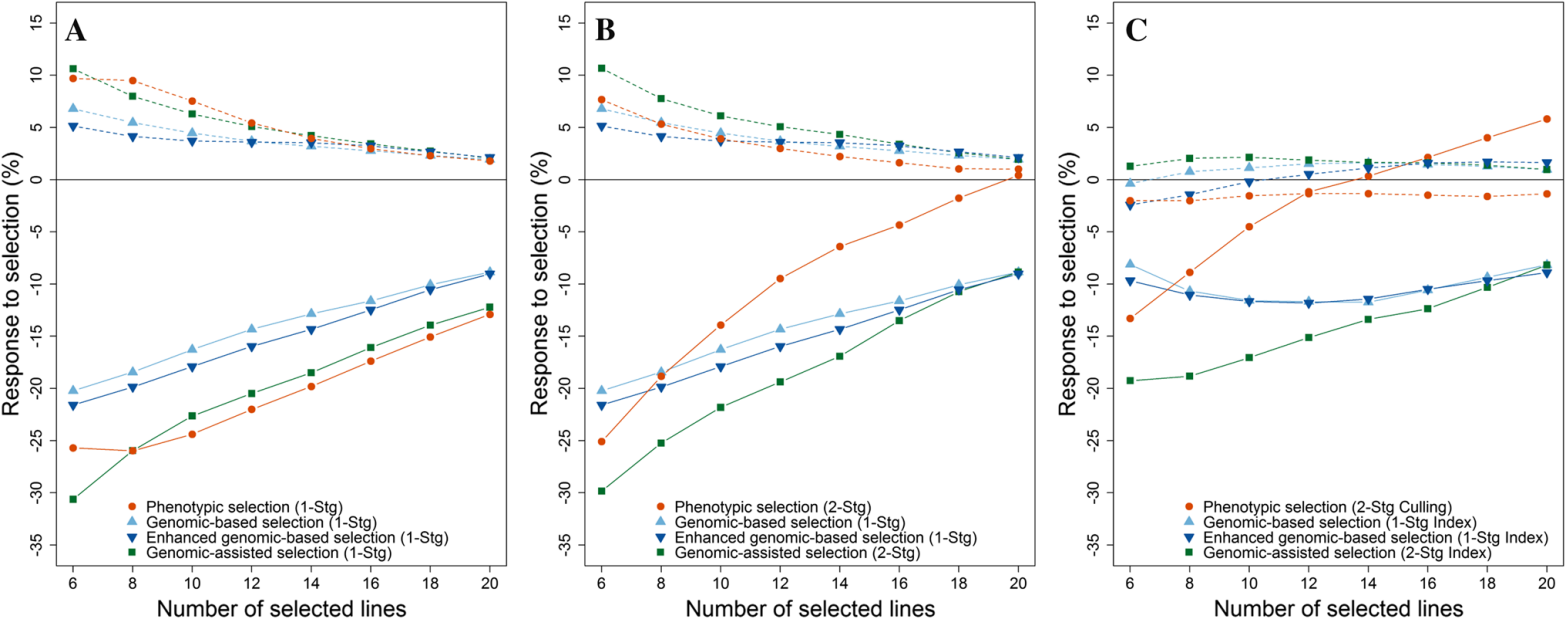
Comparison between phenotypic, genomic-based, genomic-assisted and marker-assisted selection strategies for FHB severity (solid line) and plant height (dashed line). One-stage single-trait selection with extensive disease phenotyping (**a**), two-stage single-trait selection where half of the selection candidates are discarded in the first stage by either phenotypic selection for flowering date or genomic-based selection for FHB severity (**b**) and two-stage multi-trait selection where half of the selection candidates are discarded in the first stage either by phenotypic culling for plant height or genomic index selection (**c**)

Given the large advantage of phenotypic over genomic-based selection in terms of prediction ability (+ 40% for FHB severity), several simulated selection experiments were conducted to further test under which circumstances genomic selection could still be useful when breeding for FHB resistance in durum. The selection among a randomly sampled set of 40 lines from the validation population showed a larger response for FHB severity by phenotypic than genomic-based selection in the selection strategy with extensive disease resistance phenotyping, reflecting the higher prediction ability of the former method (Fig. 6a). Nevertheless, genome-wide marker information could also be employed to improve phenotypic selection, which led though merely for the highest selection intensity to a higher response to selection. Enhancing prediction models by integrating the marker IWB64968 on chromosome 3B as fixed effect into the prediction model gave on the other hand a constant relative advantage over the basic genomic breeding values, which was though still inferior to phenotypic selection in this one-stage single-trait selection.

The second simulated selection experiment aimed to reflect the need of a two-stage selection with preselection in many breeding programmes. Despite a lower prediction ability, the results showed a strong advantage of genomic over phenotypic selection due to earlier available information and even a genomic-based selection gave an average advantage of 43% over the two-stage phenotypic selection where no FHB phenotypic information was available in first stage (Fig. 6b). Markedly, a genomic-based preselection of lines followed by genomic-assisted selection in the disease nursery showed a relatively seen 80% higher response to selection than feasible by phenotypic selection. The indirect response to selection for flowering date was negligible as expected by the low phenotypic correlation with FHB severity, underlining its role as a ‘neutral trait’ in this study (Fig. S2B). The indirect response to selection for plant height was on the other hand quite eminent for all selection methods, which would result in the selection for taller plants when aiming to increase FHB resistance due to the negative correlation between FHB severity and plant height (Fig. 6b).

Employing a genomic selection index was able to compensate for this undesirable indirect response to plant height; it resulted though also in a lower response to selection for FHB severity (Fig. 6c). Phenotypic culling in the first stage of the two-stage selection strategy for plant height resulted even in a decrease below the population average for this trait; however, a pronounced response to FHB severity was merely achieved for selection intensities larger than 30%, i.e. 12 out of 40 lines. Genomic-based index selection among all 40 selection candidates with advancing merely the most promising lines for testing in a disease nursery followed by a genomic-assisted index selection in the second stage showed finally the highest performance among all methods in this multi-trait two-stage selection scenario. Nevertheless, such a preselection resulted though in slightly less response in comparison with extensive phenotyping highlighting both the potential and limitations of a genomic-based preselection.

## Discussion

### A major QTL for FHB resistance in durum wheat on chromosome 3BS

An international collection of 228 cultivars and breeding lines was evaluated under high FHB disease pressure in artificially inoculated and humidity controlled field trials over three consecutive years to further explore the genetic basis of FHB resistance in the elite durum wheat gene pool. Although a lack of highly resistant material was evident, FHB severity levels showed a broad variation—between moderately resistant, as revealed for the North Dakota variety ‘Belzer’ with a disease severity of about 35% diseased spikelets and highly susceptible with 90% infected spikelets for the Spanish line ‘Artena’. Plant height strongly influenced FHB severities and genome-wide association mapping detected the two main contributors on chromosome 5A and the semi-dwarfing gene *Rht*-*B1b*. The 5A plant height QTL mapped in proximity of the gibberellin-responsive dwarfing gene *Rht12* originating from the gamma ray-induced winter wheat mutant ‘Karcagi 522M7K’ (Viglasi 1968). *Rht12* is located distally on the long arm of chromosome 5A, approximately 5.4 cM from locus *Xwms291* (Korzun et al. 1997) and is most likely the origin of the identified plant height QTL on chromosome 5A, as, moreover, some cultivars, for example, ‘Italo’, are known to possess *Rht12* (M. Maccaferri, unpublished). Plant height is one of the foremost morphological traits impairing FHB severity, with the general dependency—the shorter the plants the more severe are FHB epidemics, resulting in numerous co-localizations of plant height and resistance QTL (Mesterhazy 1995; Hilton et al. 1999; Buerstmayr et al. 2009; Mao et al. 2010). The widely deployed semi-dwarfing alleles *Rht*-*D1b* and *Rht*-*B1b* have been frequently found to be associated with increased susceptibility, and this negative trade-off caused by *Rht*-*B1b* could also be confirmed in durum wheat (Buerstmayr et al. 2012; Prat et al. 2017; Miedaner et al. 2017). The recently identified dwarfing gene Rht24 on chromosome 6A reduces plant height without adverse effects on FHB resistance providing a valuable source for FHB resistance breeding programmes (Herter et al. (2018).

Despite the confounding effect of putative resistance QTL with plant height, one high-confidence QTL on chromosome 3B that had a minimal effect on plant height and flowering date could be identified in the study at hand. Its additive effect corresponded to approximately 1 scoring point on a 1–9 scale, which was rather large considering an estimated range between 5 and 9 scoring points of the entire population in this study. This major resistance QTL was positioned on the short arm of chromosome 3B in close proximity to the prominent resistance QTL *Fhb1* found in hexaploid wheat cultivars, e.g. Sumai-3, Wangshuibai and derivatives (Anderson et al. 2001; Buerstmayr et al. 2009). Interestingly, also in other durum wheat lines resistance-improving alleles were found to coincide with the *Fhb1* interval. Association mapping in Tunisian-derived durum populations revealed FHB resistance QTL in the above-mentioned region (Ghavami et al. 2011), and in a population of the highly resistant *Triticum dicoccum* line ‘Td161’ with the susceptible Austrian durum cultivar Floradur, the durum cultivar contributed a resistance allele mapping in the *Fhb1* region (Buerstmayr et al. 2012). The identity of these resistance QTL detected in durum wheat in the *Fhb1* QTL interval is likely, and our study underlined the importance of the specific region in a broad durum wheat collection with 35% of the durum lines carrying the resistant allele. By now the complete contig sequence of the *Fhb1* region has been established demonstrating that the QTL interval deviates from the Chinese Spring reference in DNA size and gene content with several genes unique for the *Fhb1* donors (Rawat et al. 2016; Schweiger et al. 2016). Among these unique genes, a pore-forming toxin-like (PFT) gene was identified as the causal gene behind FHB resistance (Rawat et al. 2016). Haplotype analysis of the durum panel proposed other gene function behind the 3B QTL detected in durum wheat as the PFT gene was only present in two of the 228 analysed durum lines. Notably, only the susceptible Syrian landrace ‘Haurani’ possessed the ‘resistant’ PFT haplotype that is in contrast to a study of 40 wheat landraces and cultivars where the PFT allele was exclusively found in FHB resistant lines (Rawat et al. 2016). In a broader study of 348 hexaploid wheat accessions of mainly Chinese origin, the ‘resistant’ PFT allele existed also in susceptible accessions (He et al. 2018) and in a collection of 151 cultivars 44 lines were positive for the Wangshuibai/Sumai-3 PFT allele, but only 12 of them were resistant (Jia et al. 2018). *Fhb1* has been introgressed in hexaploid wheat and durum wheat, mostly efficiently improved FHB resistance levels but the recurrent parent had a large impact on the effect of *Fhb1* suggesting a large dependency on the genetic background (Von der Ohe et al. 2010; Salameh et al. 2011; Balut et al. 2013; Prat et al. 2017). However, the role of PFT on FHB resistance is under discussion, as Jia et al. (2018) claimed that association of PFT with resistance in some germplasm is due to its tight linkage to the actual unknown resistance gene.

The second high-confidence FHB resistance QTL was positioned in the telomeric region of chromosome 6BL dissimilar to the well-known FHB resistance QTL *Fhb2* (Anderson et al. 2001) and to other resistance genes identified on chromosome 6B in tetraploid wheat (Somers et al. 2006; Buerstmayr et al. 2012, 2013) as well as in hexaploid wheat (Bonin and Kolb 2009; Zhang et al. 2010; Basnet et al. 2012; Szabó-Hevér et al. 2014; Buerstmayr and Buerstmayr 2015). Most importantly, especially the identified resistance QTL on chromosome 3BS denotes a valuable finding for resistance breeding as the variation for FHB resistance is very limited in the primary gene pool of durum wheat. The associated SNP marker IWB64968 is also included in the 15K wheat SNP array allowing fast and cost-efficient implementation in breeding programmes, the conversion of the polymorphism in an user-friendly KASP marker can further facilitate the deployment of the found marker–trait association. Although this suggests some merit of utilizing the QTL for improving FHB resistance in durum, further validation in other genetic backgrounds is certainly needed, e.g. their segregation and effect in recent elite germplasm, before an application can be recommended in durum breeding programmes.

### Improving FHB resistance in durum by genomic breeding in early generations

Some genetic progress for FHB resistance has been achieved by phenotypic selection in the past (Fuentes et al. 2005), and moderately resistant varieties are available nowadays (Clarke et al. 2010; Miedaner and Longin 2014). Genomic-based selection is though an interesting alternative to costly and time-consuming scorings in disease nurseries and resulted in a relative superiority of 21% in terms of prediction ability for FHB resistance when compared with conventional marker-assisted selection in the study at hand. Marker-assisted selection with few significant markers can furthermore lead to effects of hitchhiking at the marker loci thus increasing the rate of inbreeding in comparison with genomic-based selection (Daetwyler et al. 2007; Pedersen et al. 2010; Sonesson et al. 2012), but most importantly a fixation of the favourable alleles would not allow any further genetic gain after a couple of selection cycles in a genomic breeding strategy (Miedaner et al. 2017). Prediction ability for genomic-based selection was furthermore similar as reported in previous studies focusing on genomic selection for FHB resistance in hexaploid wheat (Rutkoski et al. 2012; Mirdita et al. 2015; Arruda et al. 2016; Jiang et al. 2017), while it could be slightly enhanced (+ 8%) by modelling the found marker–trait association on chromosome 3B as fixed effect in a W-BLUP model by using the de novo GWAS approach suggested by Spindel et al. (2016).

A further option to improve the accuracy of genomic breeding values would be the usage of multi-trait genomic prediction models with pre-existing information of plant height and flowering date that could be measured earlier, easier or more cost-efficient than the main trait of interest, i.e. FHB resistance. This method has already shown some potential in the pathosystem bread wheat *Fusarium* for predicting the costly to assess mycotoxin content with prior information about FHB severity (Rutkoski et al. 2012). The correlation between FHB severity and flowering date is admittedly strongly influenced by climatic conditions such as the temperature during inoculation, which could consequently mask the true FHB resistance of some breeding lines (Emrich et al. 2008). This was, however, not an issue in this study, where the correlation between flowering date and FHB severity was low and additionally varied between years. The flowering date has also shown a low potential to improve the prediction of FHB severity in trait-assisted selection in this and a previous study (Schulthess et al. 2018), suggesting only a casual relationship between both traits without any or a very narrow genetic base. The negative correlation for plant height was also evident in the analysed durum population and resulted in the selection of taller plants when intending to increase FHB resistance both under phenotypic and genomic selection. The applied genomic selection index was a practicable option to address this issue and kept the population average for plant height stable, thus avoiding the negative trade-off yet at the cost of a lower response to selection for FHB resistance. Using pre-existing information about plant height had though also shown some value to improve the prediction accuracy for FHB severity in a trait-assisted selection, it can albeit be seen as an adjustment of FHB severity predictions to follow the underlying negative relationship and as a result it increased the negative trade-off in the multi-trait predictions. Culling for plant height or the mentioned index selection might therefore be necessary afterwards (Schulthess et al. 2018), which can, however, be expected to compensate the higher accuracy of a trait-assisted selection to some extent. On the other hand, an increase in plant height might be acceptable if minor genes for straw strength are concomitantly selected in order to prevent an increased lodging susceptibility. Hence, further studies are needed to investigate the actual merit of using early information about plant height in multi-trait prediction models for FHB resistance.

Irrespective of such model fine-tuning, the response to genomic selection is anticipated to be higher than by classical phenotypic selection as information about FHB severity could be made available sooner and support selection decisions in an earlier stage during variety development. The potential of such a strategy in durum breeding was indicated by using fixed lines in this study, although it should be mentioned that in applied breeding programmes the degree of heterozygosis will be much higher in early generation selection candidates and the genetic diversity in most cases lower than in the investigated worldwide durum collection. The accompanied improvement in the two-stage simulated selection experiments seemed nevertheless promising for practical applications. It combined previous available data from multiple phenotyping steps gave genomic breeding values that are based on multi-environment data that corresponded to records from several years. Pure phenotypic selection uses in contrast initially only 1-year data and sometimes 2-year data when selected lines are retested and will accordingly have a lower correlation with the true breeding value of a potential varietal candidate. Combining already available phenotypic records for achieving the desired selection goal of higher FHB resistance without a too large indirect response for plant height was also feasible with such a two-stage genomic selection strategy. Aside from culling, the usage of a genomic selection index (Ceron-Rojas et al. 2015) has been shown to be a convenient option for accomplishing this goal in the simulated selection experiments. A combination of both selection methods is likewise imaginable, e.g. culling outlier lines that already showed high lodging in early generations and subsequent index selection among the leftover lines to support the identification of lines with a desired trait combination. Lastly, it should be noticed that a genetically diverse training population was employed for the genomic predictions in this study, and although no clear population structure could be found, several intermixed subpopulations were identified that slightly differentiated the origin of the lines. Employing such combined training populations with multiple subpopulations can also be of interest for increasing the training population size of small subpopulations as well as obtaining information about line performances across breeding programmes, e.g. when using molecular marker data for the planning of crosses.

Apart from all its benefits, the costs for genotyping a breeding line to apply genomic selection are comparatively higher than getting one phenotype record for FHB resistance in a disease nursery (Lorenz et al. 2012; Sallam and Smith 2016). The largest advantage of genomic over phenotypic selection is most likely given when it is implemented at an early breeding stage, where phenotypic selection for the trait of interest is not feasible or its reliability very limited (Longin et al. 2015; Marulanda et al. 2016; Gaynor et al. 2017). Several thousand lines are generally remaining for testing in a medium-sized breeding programme at this stage, which would imply a huge effort to derive phenotypic data for FHB severity in such cases, especially as replicated testing in several locations is usually necessary to obtain reliable results (Fuentes et al. 2005). However, a lot of these lines will be discarded due to insufficient performance for other traits than FHB resistance that might be among others associated with their general field impression. This will, however, make a substantial number of data points generated in a FHB nursery obsolete beforehand. On the other hand, only these preselected lines are usually genotyped when implementing genomic selection in a line breeding programme at this stage, which allows finding a resource allocation where costs between genotyping and extensive early disease phenotyping balance-out. The additional genomic breeding values that could be derived for traits related to yield and quality aside from disease resistance would almost certainly prove to be another valuable resource for supporting selection decisions in early generations (He et al. 2016; Thorwarth et al. 2017; Hayes et al. 2017; Fiedler et al. 2017).

Notwithstanding that FHB resistance is an important trait in durum breeding (Prat et al. 2014; Clarke et al. 2010), it cannot be the only selection criterion and first eliminating the lines most susceptible to FHB followed by selection for other agronomical important traits might thus be an appropriate strategy. Another yet complementary option to reduce the phenotyping intensity would be a stricter selection and advancing fewer lines to the next breeding stage, i.e. for testing in multi-environment trials, given that they are more promising than the ones identified by early generation phenotypic selection (Michel et al. 2017). Such lines would also be interesting crossing parents, whose genomic fingerprints can be aside from the above-discussed product development further used in the planning of crosses with genome-wide marker data to target a general population improvement of the entire germplasm in a breeding programme (Zhong and Jannink 2007; Akdemir and Sánchez 2016; Lehermeier et al. 2017; Osthushenrich et al. 2017; Müller et al. 2018).

## Conclusions

Comparison between phenotypic and genomic selection for FHB resistance revealed a superior prediction ability of the former; nevertheless, simulated selection experiments resulted in a higher response to selection when using genomic breeding values for an early generation selection and focused disease phenotyping of a preselected set of the most promising lines. The usage of genome-wide marker data for genomics-assisted selection can thus be a valuable tool for supporting breeders in their selection decisions by making germplasm information earlier accessible and more reliable. Increasing the frequency of favourable alleles at major QTL by upweighting their effect in genomic prediction models can thereby be an important aspect as shown for the QTL identified on chromosome 3BS for FHB resistance in this study. Prior knowledge of trait genetic architecture and basic genomic breeding values could together be used for either positive or negative tandem, culling or index selection within and across families for specific traits and trait complex in combination with breeder’s knowledge about parents, plant material and breeding goals. The genetic progress for FHB resistance in the elite durum gene pool could accordingly be accelerated in the short term with such a strategy, while sources of resistance from exotic germplasm could serve to broaden the genetic base for FHB resistance beyond the capabilities of elite material for achieving higher levels of FHB resistance in durum wheat in the long-term.

### Author contribution statement

BS and SM wrote the manuscript and analysed the data. BS, HB, ML designed the field trials and collected the phenotypic data. MM, RT and HB initiated and guided through the study. All authors read and approved the final manuscript.

## Electronic supplementary material

Below is the link to the electronic supplementary material.

Supplementary material 1 (PDF 172 kb)

Supplementary material 2 (PDF 193 kb)

Supplementary material 3 (PDF 183 kb)

Supplementary material 4 (PDF 171 kb)

Supplementary material 5 (DOCX 18 kb)

Supplementary material 6 (XLSX 53 kb)

Supplementary material 7 (DOCX 27 kb)
