# Supplementary material for: Exploring and exploiting the genetic variation of Fusarium head blight resistance for genomic-assisted breeding in the elite durum wheat gene pool
Source: Theor Appl Genet. 2018 Dec 1;132(4):969–88. doi: 10.1007/s00122-018-3253-9 (PMC6449325; doi:10.1007/s00122-018-3253-9)
Supplement: Supplementary file 1 — Supplementary material 1 (PDF 172 kb) [file 122_2018_3253_MOESM1_ESM.pdf]

Figure S1

Article Title: Exploring and exploiting the genetic variation of Fusarium head blight resistance for genomic-assisted breeding in the elite durum wheat gene pool

Journal: Theoretical and Applied Genetics

Authors: Barbara Steiner, Sebastian Michel, Marco Maccaferri, Marc Lemmens, Roberto Tuberosa, Hermann Buerstmayr

Name, affiliation, and email of corresponding author:

Sebastian Michel
Department for Agrobiotechnology (IFA-Tulln)
Institute for Biotechnology in Plant Production
University of Natural Resources and Life Sciences, Vienna (BOKU)
Konrad-Lorenz-Str. 20, 3430 Tulln, Austria
e-mail: sebastian.michel@boku.ac.at

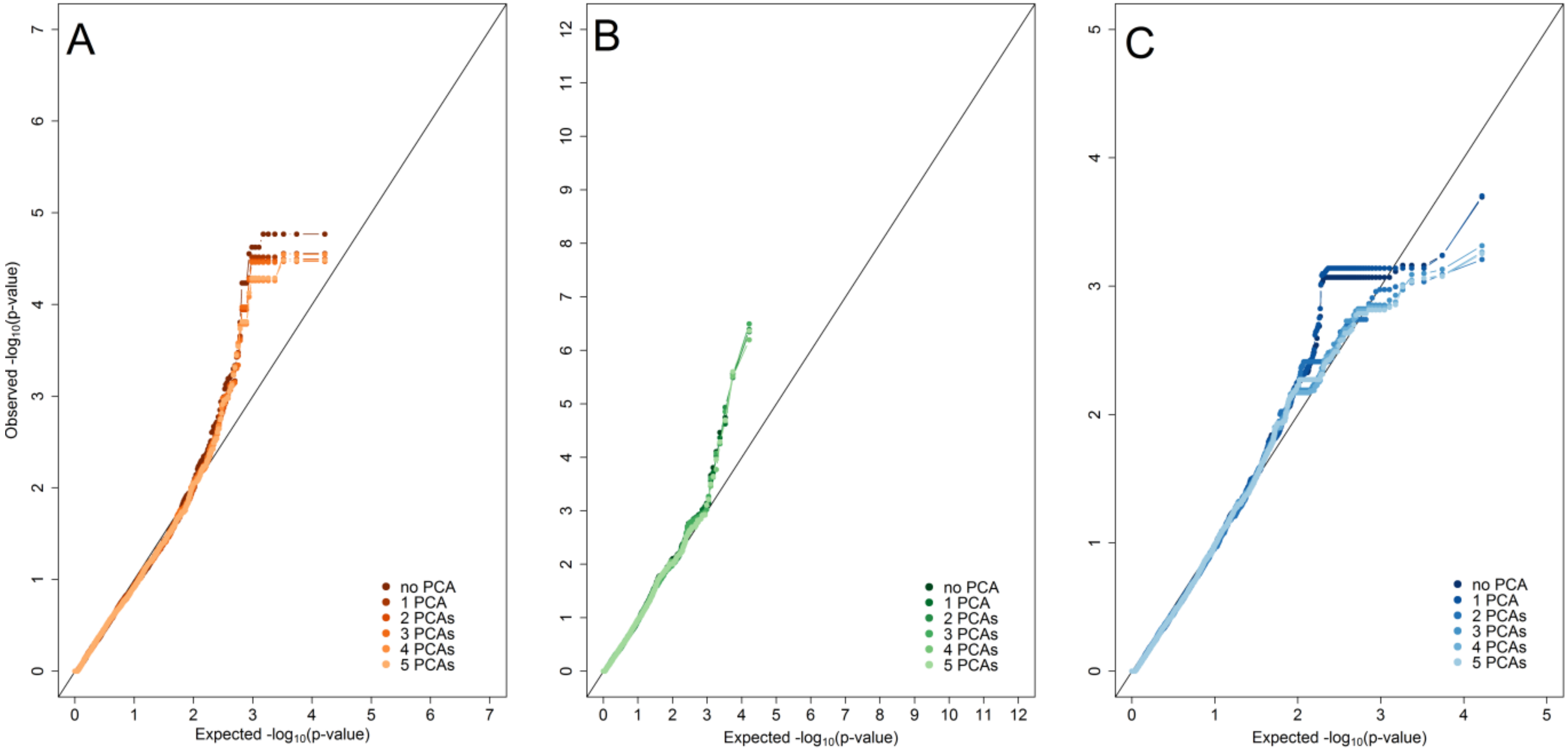

Fig. S1 Quantile-quantile plot of the expected versus observed $-\log_{10}$ (P) values for FHB severity (A), plant height (B), and flowering date (C) modelling none or the 1-5 first principal components as fixed effect in the mixed model employed for genome-wide association mapping additional to correcting kinship by a genomic relationship matrix
