# Supplementary material for: Exploring and exploiting the genetic variation of Fusarium head blight resistance for genomic-assisted breeding in the elite durum wheat gene pool
Source: Theor Appl Genet. 2018 Dec 1;132(4):969–88. doi: 10.1007/s00122-018-3253-9 (PMC6449325; doi:10.1007/s00122-018-3253-9)
Supplement: Supplementary file 4 — Supplementary material 4 (PDF 171 kb) [file 122_2018_3253_MOESM4_ESM.pdf]

Figure S4

Article Title: Exploring and exploiting the genetic variation of Fusarium head blight resistance for genomic-assisted breeding in the elite durum wheat gene pool

Journal: Theoretical and Applied Genetics

Authors: Barbara Steiner, Sebastian Michel, Marco Maccaferri, Marc Lemmens, Roberto Tuberosa, Hermann Buerstmayr

Name, affiliation, and email of corresponding author:

Sebastian Michel
Department for Agrobiotechnology (IFA-Tulln)
Institute for Biotechnology in Plant Production
University of Natural Resources and Life Sciences, Vienna (BOKU)
Konrad-Lorenz-Str. 20, 3430 Tulln, Austria
e-mail: sebastian.michel@boku.ac.at

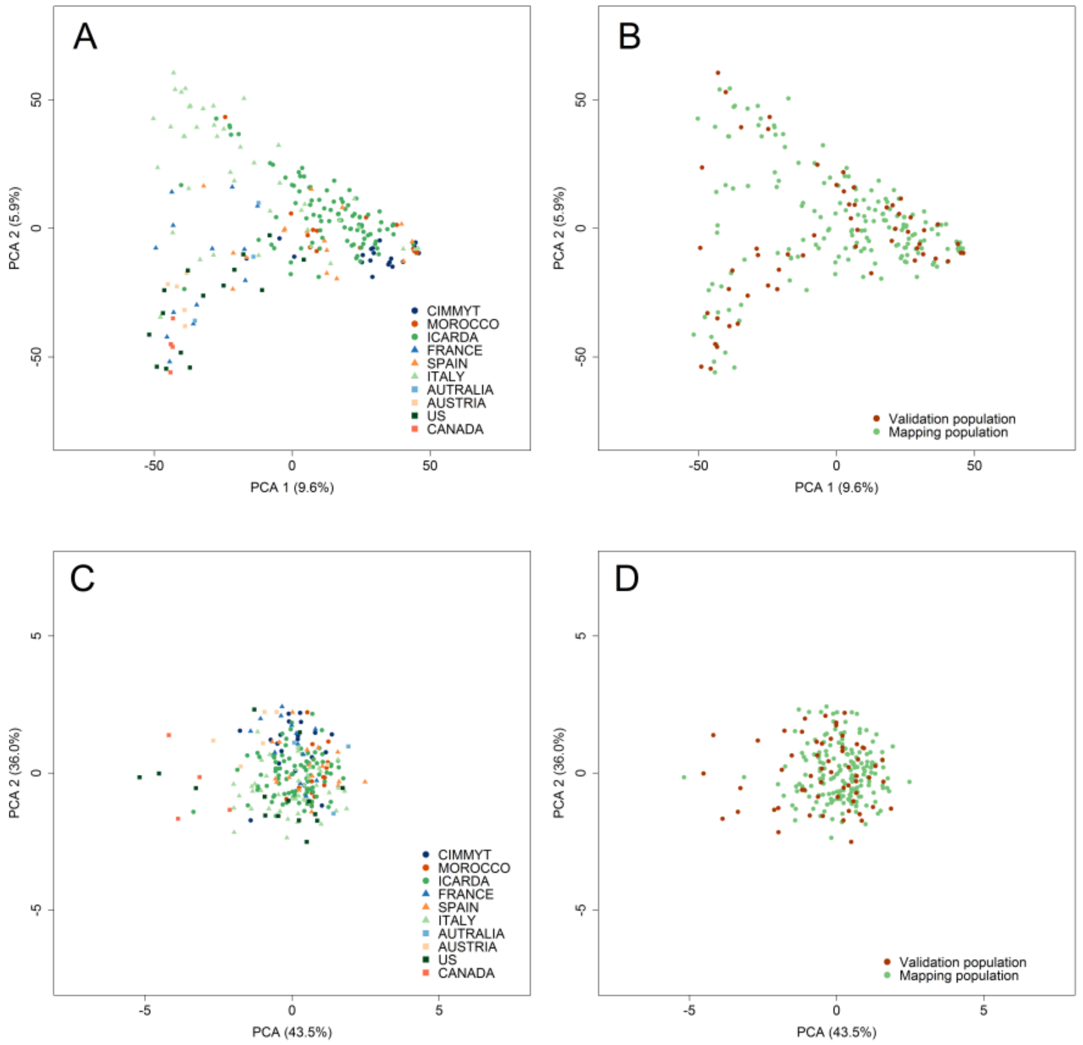

Fig. S4 Population structure as determined by molecular markers highlighting the different origins of lines (A) and the relationship between the mapping and validation population (B) as well the corresponding phenotypic differentiation based on plant height, flowering date, and FHB severity (C and D)
