# Supplementary material for: Exploring and exploiting the genetic variation of Fusarium head blight resistance for genomic-assisted breeding in the elite durum wheat gene pool
Source: Theor Appl Genet. 2018 Dec 1;132(4):969–88. doi: 10.1007/s00122-018-3253-9 (PMC6449325; doi:10.1007/s00122-018-3253-9)
Supplement: Supplementary file 5 — Supplementary material 5 (DOCX 18 kb) [file 122_2018_3253_MOESM5_ESM.docx]

**Table S1**

**Article Title:** Exploring and exploiting the genetic variation of Fusarium head blight resistance for genomic-assisted breeding in the elite durum
wheat gene pool

**Journal**: Theoretical and Applied Genetics

**Authors**: Barbara Steiner, Sebastian Michel, Marco Maccaferri, Marc Lemmens, Roberto Tuberosa, Hermann Buerstmayr

**Name, affiliation, and email of corresponding author:**

Sebastian Michel

Department for Agrobiotechnology (IFA-Tulln)

Institute for Biotechnology in Plant Production

University of Natural Resources and Life Sciences, Vienna (BOKU)

Konrad-Lorenz-Str. 20, 3430 Tulln, Austria

e-mail: sebastian.michel@boku.ac.at

**Table S1** Correlations between Fusarium head blight severity (FHB), plant height (PH) and flowering date (FD) for the individual trials and across the entire trial series 2011-2013.

|  | FHB 2012 | FHB 2013 | FD 2011 | FD 2012 | FD 2013 | PH 2011 | PH 2012 | PH 2013 | FHB | FD | PH |
| --- | --- | --- | --- | --- | --- | --- | --- | --- | --- | --- | --- |
| FHB 2011 | 0.54 | 0.53 | 0.18 | 0.02 | 0.02 | -0.31 | -0.23 | -0.27 | 0.86 | 0.07 | -0.28 |
| FHB 2012 |  | 0.44 | -0.03 | -0.07 | -0.09 | -0.20 | -0.15 | -0.17 | 0.74 | -0.10 | -0.19 |
| FHB 2013 |  |  | 0.19 | 0.06 | 0.14 | -0.27 | -0.21 | -0.20 | 0.82 | 0.15 | -0.24 |
| FD 2011 |  |  |  | 0.51 | 0.51 | 0.00 | 0.11 | 0.02 | 0.17 | 0.77 | 0.05 |
| FD 2012 |  |  |  |  | 0.59 | 0.15 | 0.27 | 0.08 | 0.04 | 0.83 | 0.19 |
| FD 2013 |  |  |  |  |  | 0.17 | 0.19 | 0.11 | 0.06 | 0.87 | 0.17 |
| PH 2011 |  |  |  |  |  |  | 0.79 | 0.79 | -0.32 | 0.14 | 0.92 |
| PH 2012 |  |  |  |  |  |  |  | 0.82 | -0.23 | 0.24 | 0.94 |
| PH 2013 |  |  |  |  |  |  |  |  | -0.26 | 0.10 | 0.92 |
| FHB |  |  |  |  |  |  |  |  |  | 0.09 | -0.29 |
| FD |  |  |  |  |  |  |  |  |  |  | 0.19 |
