# Supplementary material for: Exploring and exploiting the genetic variation of Fusarium head blight resistance for genomic-assisted breeding in the elite durum wheat gene pool
Source: Theor Appl Genet. 2018 Dec 1;132(4):969–88. doi: 10.1007/s00122-018-3253-9 (PMC6449325; doi:10.1007/s00122-018-3253-9)
Supplement: Supplementary file 7 — Supplementary material 7 (DOCX 27 kb) [file 122_2018_3253_MOESM7_ESM.docx]

**Table S3**

**Article Title:** Exploring and exploiting the genetic variation of Fusarium head blight resistance for genomic-assisted breeding in the elite durum
wheat gene pool

**Journal**: Theoretical and Applied Genetics

**Authors**: Barbara Steiner, Sebastian Michel, Marco Maccaferri, Marc Lemmens, Roberto Tuberosa, Hermann Buerstmayr

**Name, affiliation, and email of corresponding author:**

Sebastian Michel

Department for Agrobiotechnology (IFA-Tulln)

Institute for Biotechnology in Plant Production

University of Natural Resources and Life Sciences, Vienna (BOKU)

Konrad-Lorenz-Str. 20, 3430 Tulln, Austria

e-mail: sebastian.michel@boku.ac.at

**Table S3** Chromosomal position, QTL detection frequency, and additive effect of markers associated with Fusarium head blight severity (FHB), plant height (PH) and flowering date (FD) in the mapping and validation populations as well as in the entire population of 228 lines.

|  |  |  |  |  | Add. eff. population^§^ | | |  | Add. eff. mapping^¶^ | | |  | Add. eff. validation^#^ | | |  |  |
| --- | --- | --- | --- | --- | --- | --- | --- | --- | --- | --- | --- | --- | --- | --- | --- | --- | --- |
| Marker | Chr. | Pos. | Trait^†^ | p^‡^ | FHB | PH | FD |  | FHB | PH | FD |  | FHB | PH | FD | FA^††^ | ρ_G_^‡‡^ |
| IWB72690 | 1A | 1.7 | FHB | 3.15 | -23.8 | -1.3 | -0.2 |  | -30.0 | -1.1 | -0.2 |  | -1.6 | -1.7 | -0.1 | 0.29 | 9.7 |
| IWB36357 | 1B | 82.7 | FD | 3.11 | 18.3 | 0.6 | -0.5 |  | 18.5 | 0.4 | -0.6 |  | 25.0 | 1.4 | -0.3 | 0.89 | 5.3 |
| IWB32396 | 2A | 101.6 | FD | 3.16 | -47.9 | 0.3 | -0.8 |  | -48.2 | 0.2 | -0.8 |  | -48.8 | 0.8 | -0.7 | 0.86 | 19.1 |
| IWB46663 | 2A | 109 | FHB | 3.23 | -67.6 | 0.9 | 0.2 |  | -63.2 | 0.6 | 0.1 |  | -80.3 | 1.9 | 0.1 | 0.32 | 1.8 |
| IWB44254 | 2A | 158.7 | PH | 3.14 | 29.2 | -1.1 | 0.4 |  | 32.3 | -1.2 | 0.3 |  | 78.0 | -4.5 | 0.9 | 0.88 | 0.5 |
| IWB36028 | 2A | 181.2 | PH | 4.47 | 70.2 | -2.6 | 0.0 |  | 58.1 | -2.3 | 0.0 |  | 95.0 | -2.9 | 0.1 | 0.87 | 4.6 |
| IWB24986 | 2A | 197.6 | FHB | 3.8 | -84.9 | 1.4 | 0.1 |  | -88.4 | 0.7 | -0.1 |  | -72.4 | 2.3 | 0.4 | 0.13 | 5.7 |
| IWB40861 | 2B | 53.4 | FD | 3.07 | 21.5 | -0.9 | -0.6 |  | 8.5 | -0.6 | -0.5 |  | 63.9 | -1.9 | -0.6 | 0.57 | 11.9 |
| IWB5439 | 2B | 172.3 | FHB | 3.3 | -52.7 | 1.6 | 0.1 |  | -47.1 | 1.1 | 0.2 |  | -71.0 | 3.3 | -0.2 | 0.26 | 4.9 |
| IWB64968 | 3B | 8.0 | FHB | 4.55 | -65.8 | 0.8 | 0.0 |  | -61.5 | 0.3 | -0.1 |  | -74.7 | 1.8 | 0.1 | 0.35 | 17.8 |
| IWB36517 | 3B | 92.2 | PH | 3.14 | 8.9 | -1.4 | -0.1 |  | 7.7 | -1.3 | -0.2 |  | 14.9 | -1.7 | 0.1 | 0.24 | 7.8 |
| IWB24360 | 4A | 105.5 | FD | 3.02 | -7.3 | -0.8 | -0.7 |  | -7.2 | -1.0 | -0.6 |  | -20.7 | 0.7 | -0.9 | 0.11 | 5.4 |
| IWB74227 | 4B | 2.8 | FD | 3.06 | 42.9 | -1.7 | -0.7 |  | 47.1 | -1.2 | -0.7 |  | 26.2 | -2.7 | -0.7 | 0.87 | 10.3 |
| IWB56078 | 4B | 32.9 | PH | 5.56 | 32.1 | -2.1 | 0.0 |  | 25.6 | -1.9 | -0.1 |  | 48.0 | -2.7 | 0.3 | 0.81 | 11.3 |
| IWA1670 | 5A | 188.9 | PH | 4.74 | 49.7 | -2.3 | 0.3 |  | 47.2 | -1.7 | 0.3 |  | 61.4 | -4.4 | 0.0 | 0.81 | 8.4 |
| IWB70133 | 6A | 124.8 | FHB | 3.19 | -42.3 | -0.4 | -0.4 |  | -52.9 | 0.0 | -0.4 |  | -14.3 | -1.7 | -0.4 | 0.83 | 3.8 |
| IWB66697 | 6B | 155.1 | FHB | 4.62 | -64.9 | 1.5 | 0.1 |  | -79.3 | 1.8 | 0.1 |  | -1.4 | 0.1 | 0.2 | 0.11 | 9.0 |

^†^ The trait for which the marker was detected in the mapping population

^‡^ -log_10_(p-value) with high confidence marker-trait associations being underlined

^§^ Additive effect in the entire population of 228 lines

^¶^ Additive effect in the mapping population

^#^ Additive effect in the validation population

^††^ Frequency of lines carrying the favourable allele (%)

^‡‡^ Explained genetic variance (%)
